# Defining functional distances over Gene Ontology

**DOI:** 10.1186/1471-2105-9-50

**Published:** 2008-01-25

**Authors:** Angela del Pozo, Florencio Pazos, Alfonso Valencia

**Affiliations:** 1 Structural Biology and Biocomputing Programme, Spanish National Cancer Research Centre (CNIO), Melchor Fernandez Almagro, 3. E-28029 Madrid, Spain; 2 Computational Systems Biology Group, National Biotechnology Centre (CNB-CSIC). Darwin 3, 28049, Madrid, Spain

## Abstract

**Background:**

A fundamental problem when trying to define the functional relationships between proteins is the difficulty in quantifying functional similarities, even when well-structured ontologies exist regarding the activity of proteins (i.e. 'gene ontology' -GO-). However, functional metrics can overcome the problems in the comparing and evaluating functional assignments and predictions. As a reference of proximity, previous approaches to compare GO terms considered linkage in terms of ontology weighted by a probability distribution that balances the non-uniform 'richness' of different parts of the Direct Acyclic Graph. Here, we have followed a different approach to quantify functional similarities between GO terms.

**Results:**

We propose a new method to derive 'functional distances' between GO terms that is based on the simultaneous occurrence of terms in the same set of Interpro entries, instead of relying on the structure of the GO. The coincidence of GO terms reveals natural biological links between the GO functions and defines a distance model *D*_*f *_which fulfils the properties of a Metric Space. The distances obtained in this way can be represented as a hierarchical 'Functional Tree'.

**Conclusion:**

The method proposed provides a new definition of distance that enables the similarity between GO terms to be quantified. Additionally, the 'Functional Tree' defines groups with biological meaning enhancing its utility for protein function comparison and prediction. Finally, this approach could be for function-based protein searches in databases, and for analysing the gene clusters produced by DNA array experiments.

## Background

Current genome sequencing projects are producing a wealth of data in the form of sequences of biological polymers. For this data to be useful, it has to be interpreted in functional terms. Thus, efficient systems to describe and classify protein function are needed, as well as tools to predict the function of the huge number of new sequences.

There is much evidence for the need of well-defined and structured functional descriptions [1-4]. However, the main difficulty encountered is that 'function' is not a well defined concept and it is not as un-equivocal as 'sequence' or 'structure'. Indeed, protein function is a very complex and multidimensional phenomenon.

In many cases, functional descriptors are based on the available experimental techniques or are due to historical reasons. However, they do not necessarily have any meaning in biological terms (evolution, molecular mechanism). The methods we use to study biological systems require conceptualization and categorization, which are sometimes taken beyond their role as mere tools of the scientific method and are 'imposed' on the cell. One example is the artificial distinction between processes such as 'transmission of information' (for example DNA/RNA processing), 'metabolism' (of small compounds) and 'transport' (communication with the environment). Such disjointed classifications, as used in the first schemes to describe protein function, clearly do not extend to the molecular or evolutionary level. These schemes have been used in the past for classifying proteins into functional classes and for developing systems to assign newly sequenced proteins to them [5,6].

The current tendency is to use vocabularies and ontologies that allow complex functional descriptions beyond disjointed classes. Among these, the important effort of the Open Biomedical Ontologies (OBO) [7] in developing controlled vocabularies for a wide scope of applications in a biological and medical context must be recognised. The OBO ontologies are designed as graphic architectures formed by univocal concepts (terms) that are linked together by relationships that satisfy some prefixed and formal rules [8]. The Gene Ontology (GO) project [9] has become the 'de-facto' standard in biomedical ontologies. Formally, GO is designed as a Direct Acyclic Graph (DAG) based on two unconstrained relationships ('is-a' and 'part-of') that link a vocabulary of functional terms [2]. This graph structure, together with the simple conceptualization, permits comparisons between any two GO terms to assess their functional similarity. However, certain problems, such as the function-based search for potential genes/proteins of interest across multiple annotated databases and the analysis of high throughput microarray data, have led to the in depth exploration of ontology in order to propose models and criteria to measure the functional relationships between the terms.

In recent years, many studies have addressed this matter [10-14], although Lord was the first to establish a semantic distance for any two terms in GO [10], adjusting the ideas of Resnik [15] for general taxonomies. In the model proposed by Lord, the similarity of any two GO terms is determined as a function of the information content of common ancestors that are calculated from corpus statistics. Recently, further efforts to identify functionally related gene products in annotated databases based on the distances calculated by Lord [11] have been shown to produce a good agreement with homology searches [12]. Nevertheless, using the more informative common ancestors as a proximity reference presents some restrictions. First, the depth of the shared parent nodes is not a suitable criteria for some limited cases in which the terms to be compared are close to the root. Furthermore, the information content (i.e. probability) of a node is highly dependent on the annotated database selected and its release version.

Models have been developed to overcome these limitations that take into account other aspects of the ontology structure. For example, the distance between two terms may also integrate the density of the terms and the path that links them [13]. Alternatively, a new definition has been used that considers the local relationships in the subgraph generated by the terms, rather than their global positions in the DAG [14].

A common feature of these different approaches is that they rely mainly on the semantic links of the DAG. Unfortunately, there are inherent problems in this approach due to the non-homogeneity and the uneven distribution of the biological knowledge. As a result some regions of the DAG are more densely populated than others, so that the connections between terms are not comparable. In addition, the depth of a node (which is related to its specificity) can not be assigned in an unequivocal way. This type of problem is especially relevant for nodes that are profusely connected to the root by various paths of different lengths.

In this work, we propose a novel method that associates the Molecular Function GO (MF-GO) terms based on their co-occurrences in a 'curated' set of proteins and enriched by the semantic relationships from the ontology. Interpro is used as a curated database as it integrates protein information from other databases that describe protein families, domains and functional sites, such as PROSITE, PRINTS, Pfam, ProDom, SMART and TIGRFAMs [16].

Conceptually, the method is, to some extend, similar to the way in which similarities between aminoacids are 'learnt' from examples (structural curated alignments) rather than obtained from the raw chemical properties of the aminoacids. Methodologically, it shares aspects of the algorithm used in the DAVID tool [17] for clustering heterogeneous annotation contents from different resources into annotation groups based on the co-association of the annotated genes in the databases.

The method analyses the mutual occurrences of the MF-GO terms across the Interpro entries. The occurrences are used as the basis of the comparison of the terms on the assumption that the persistent coincidence of two terms describes its 'relation' in the general functional space. The analysis of the occurrences provides a useful mathematical tool to quantify the functional similarity between terms. A hierarchical tree linking the MF-GO terms is built from the similarity matrix. We termed this tree the 'Functional Tree' and it formally constitutes a Distance Model since it satisfies the ultrametric triangle inequality. In this context, the Functional Distance for a pair of terms, *D*_*f*_, is defined as the height of their least common ancestor in the 'Functional Tree'. In addition, the tree allows the GO terms to be clustered into compact and homogeneous groups with biological meaning.

We describe here how the Functional Tree was built, how the tree is clustered and the groups generated are analyzed in terms of the functions they describe. The Functional Distance *D*_*f *_derived from the Functional Tree was used to calculate the distances between pairs of yeast proteins to assess the reliability of the tree. We also compare this new metric with another based on semantic similarities.

## Results

### Algorithm

**Figure 1:**
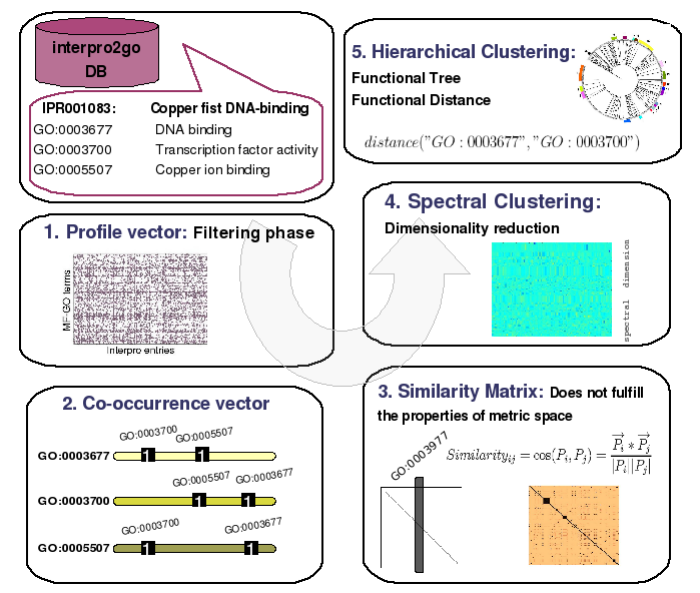
Scheme of the method used for obtaining the Metric Model based on Gene Ontology annotations. (1) Profile vectors are built by retrieving the Molecular Function Gene Ontology annotations (MF-GO terms) of Interpro domains from the file interpro2go. (2) From the profiles, a co-occurrence matrix is calculated by counting how many times two MF-GO terms occur in the same set of Interpro domains. (3) The co-occurrence vectors are feature vectors that describe the functional links of each MF-GO term. The similarity between the MF-GO terms is calculated by the cosine distance between the vectors. (4) The similarity values are arranged in a matrix *S*. The similarity matrix was considered as the Adjacency Matrix of a weighted graph *G*. The terms can be clustered by means of the partition of the graph. To obtain the best partition of *G*, a Spectral Clustering algorithm is applied. The Spectral Clustering algorithm projects the terms in a K dimensional space which can be clustered with standard clustering techniques. (5) The GO terms are grouped in a Hierarchical Tree representing the Functional Distance *D*_*f *_that satisfy the mathematical properties of a Metric Space.

The steps followed to obtain the Metric Model are schematically represented in Figure 1.

First, for each Molecular Function term we create a profile vector that represents its presence/absence in different Intepro entries (Figure 1, box 1). These vectors resemble the 'phylogenetic profiles' used to encode the proteins present in different organisms and to detect protein relationships [18,19].

Initially, we started with 1532 MF-GO terms present in 5535 Intepro entries. Additionally, we included the semantic relationships represented by the Gene Ontology DAG by assigning the same Interpro domain to the parent(s) of a given GO term. The profiles were checked to detect the terms that were associated exclusively to one Interpro entry and to ensure that this entry was not annotated with any other term. Any such profiles were removed because they do not help to extract relationships between the terms. After filtering, we obtained a matrix of 1778 Interpro entries with 1392 MF-GO terms. In a second step (Figure 1, box 2), we built a matrix of co-occurrences of GO terms in Interpro entries. The occurrences were accumulated through all the profiles and we obtained the total mutual occurrences in the universe of the 1392 terms. Each co-occurrence vector describes a MF-GO term in relation to the rest of the MF-GO terms, which enables it to be used as a feature vector in the application of statistical learning techniques.

**Figure 2:**
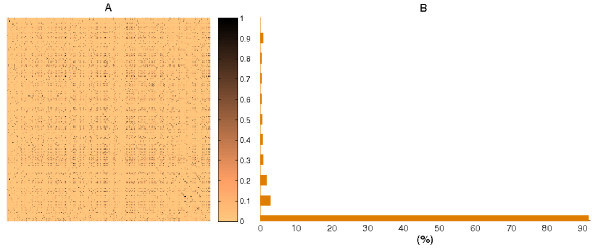
(A) Initial Similarity matrix of 1329 × 1329 dimensions. The similarity colour scale is shown at the right of the matrix. *S *is obtained from the set of co-occurrence vectors. Note that *S *is symmetric, positive, and its values are ranked between 0 and 1. (B) Distribution of the similarity values. The distribution shows that *S *is sparse and depicts a general view of the structure of the search space for the clustering of *S*.

Third, the similarity between the terms was calculated using the cosine distance between their corresponding co-occurrence vectors (Figure 1, box 3). The similarity matrix *S *was obtained by crossing the vectors all-against-all (as graphically represented in Figure 2A) and the functional groups were obtained by the clustering of *S*. Full details of the Similarity matrix calculus are available in the Methods section. Finally we applied a Spectral Clustering algorithm [20,21] as it performs a dimensional reduction of the data (Figure 1, box 4). The general ideas behind Spectral Clustering methods are introduced in the 'Spectral Clustering' subsection from the appendix. This approach improved the search for functional groups in the MF-GO terms space.

Spectral Clustering considers *S *as the Adjacency Matrix of a normalized weighted graph *G*, where the nodes stand for the MF-GO terms linked by the similarity values. Thus, the clustering problem is transformed into a partitioning graph problem. We only considered the graph comprised of terms that were connected with significant relationships, that is those connected by a pairwise similarity greater than a manually selected threshold value (see Methods, 'Similarity Matrix' subsection). After imposing this constraint, we obtained 995 MF-GO terms from the total of approximately 7500 terms integrated in the released version of this work.

**Figure 3:**
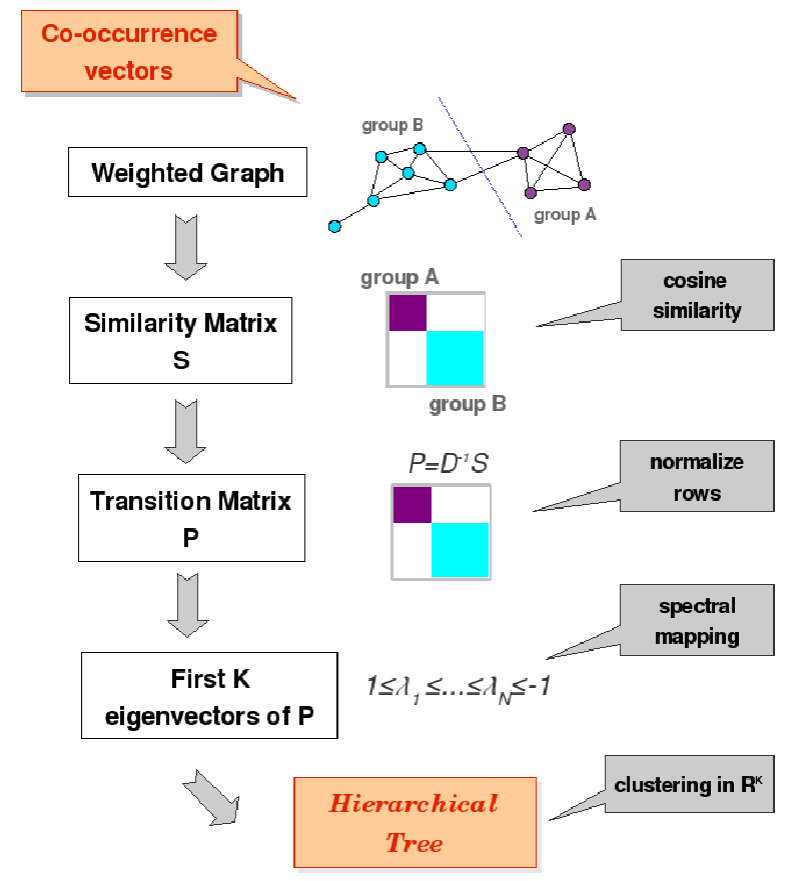
Scheme of the spectral clustering methodology. Spectral clustering techniques aim to find the best partition of a weighted graph. A graph is constructed where the nodes are MF-GO terms linked by similarity values *s*_*ij *_derived by calculating the cosine distance between the vectors of the co-occurrence matrix. The similarity matrix *S *= [*s*_*ij*_] is treated as a real-value adjacency matrix of the graph. Let *P *be a normalized matrix named the Transition Probability matrix that represents the probability of transit from one node to another in this weighted graph. *P *is calculated from *S*. The first K eigenvalues of *P *are used to map the nodes of the graph to a K-dimensional space and the points in this reduced space can be grouped by any clustering algorithm. In this work, we have applied a hierarchical clustering algorithm.

We have also considered the NJW adaptation of Spectral Clustering (NJM-SC) by Ng, Jordan and Weiss [21], which is summarized in the general scheme in Figure 3 (see the 'NJW Spectral Clustering Algorithm' subsection from the appendix). The algorithm calculates a Transition Probability Matrix, *P*, from a N × N Similarity matrix, *S*, that represents the probability of transit from one node to another in the graph. *P *is diagonalized and its K first eigenvectors are stacked and normalized in a new K × K matrix, *Y*. The rows of *Y *can be treated as N vectors K dimensional. Therefore, NJM-SC projects the MF-GO terms (nodes of *G*) onto points in a K dimensional space. Subsequently, the terms can be grouped with any standard clustering technique. K was thus selected as the number of clusters in the optimum partition of *G*. The optimization procedure is presented in detail in the Methods section. The resulting number of optimal groups was 93.

Finally, from the vector projections of the MF-GO terms, we built a dendrogram with an Agglomerative Hierarchical Clustering algorithm [22] (Figure 1, box 5). The tree obtained ('Functional Tree') defines a distance *D*_*f *_between any two MF-GO terms from the set of 995 (see Additional file 1). The distance for two terms was the minimum height of their common nodes. From a mathematical point of view, *D*_*f *_satisfies the topological properties that induces a metric space (see 'Properties of a Metric Space' from the appendix). So, the metric generated by the Functional Tree establishes a 'distance scheme' that provides a measure of the closeness of any two MF-GO terms within the tree.

### Testing

#### Functional Groups

**Figure 4:**
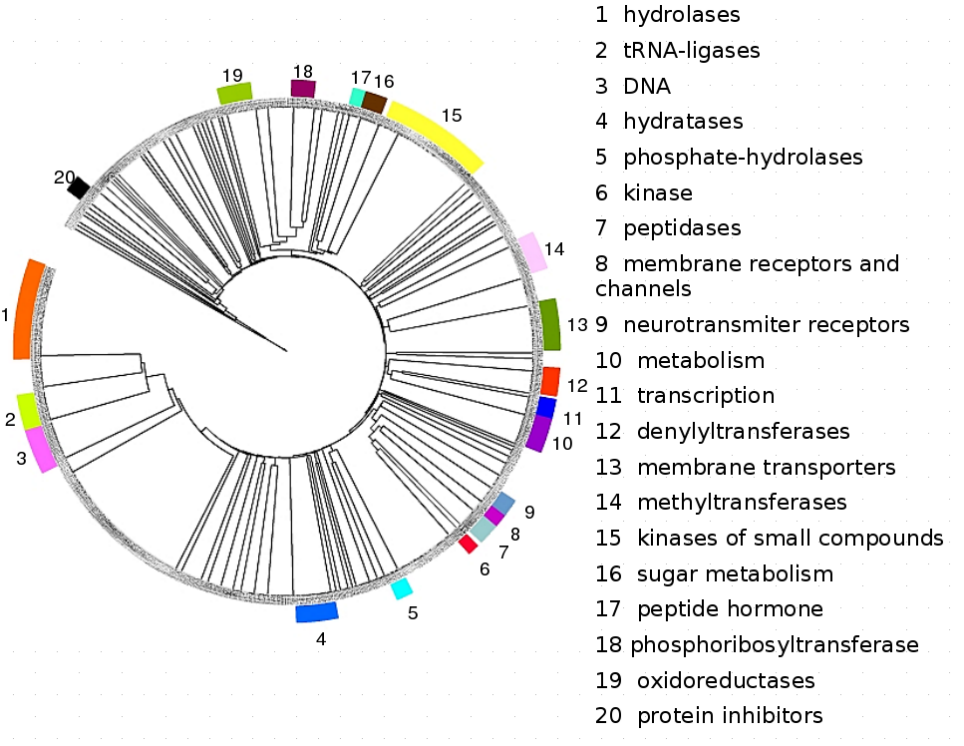
Functional Tree representation. The tree is divided into 93 groups. The groups for which a functional 'homogeneity' was qualitatively assessed are labelled and coloured over the tree. The functional labels are specified. The tree was generated with iTol [30].

The nodes of the Functional Tree are divided into groups imposing the number of clusters obtained in the optimization step. The 93 groups of Molecular Function terms are inspected and 20 groups with highly homogeneous biological function are detected. In the Functional Tree (Figure 4, see Additional file 1), the functionally homogeneous groups are coloured and ranked, and the labels assigned are shown with their rank number.

Some of these groups were very specific, like the group containing the 21 amino acyl-tRNA ligase activities. This group includes all the tRNA ligases and no other GO term, and hence the automatic clustering algorithm achieved a perfect segregation of this functional group (group 2).

Another big group mostly composed by activities related to hydrolysis (hydrolases, peptidases, nucleases, lipases) was labelled as group 1. Although this group was homogeneous for this activity, the coverage was not perfect since other hydrolases lay outside of this group. For example, group 7, which was far from group 2 in the tree, was mainly comprised of peptidase activities.

Interestingly, many different activities associated with DNA processing tended to cluster together despite the fact that they were apparently unrelated (i.e.: transcription factors and enzymes involved in DNA metabolism, DNA ligases, topoisomerases, etc... – group 3). As for the hydrolases case commented above, although this group contained only DNA-related activities and other DNA-related functional terms were not included in this group.

Most of the kinases of small metabolic compounds were clustered in a large group (group 15), while protein kinases were more widespread even though some of them clustered together in group 6. Many membrane transporters of apparently different nature (transporters for inorganic ions, drugs, proteins, etc...) were also clustered together in homogeneous groups.

All the 'protein inhibitor' activities within the dataset were clustered together in a homogeneous group (group 20), which is interesting given that the proteins they inhibit are of a very different nature (phos-phatases, ribonucleases, proteases, etc...).

Functional clustering was also evident for many other GO terms: methyl-transferases, phosphorybo-syltransferases, peptidases, some peptidic hormones, neurotransmitter receptors, phosphate-hydrolases, hy-dratases/dehydrateses, adenylyltransferases, etc....

For other clusters, this functional 'homogeneity' was not so evident. For example, oxidoreductases were spread across many groups even though some groups contained oxidoreductase activities only (group 19). This dispersion could be explained by the fact that this function is present in proteins with a very different evolutionary origin. Similarly, activities related to RNA metabolism were spread among the different clusters, except the tRNA-ligases discussed above.

In general, the clustering represented by the tree makes sense, meaning that GO molecular functions that are intuitively 'similar' were close in the tree and vice versa. This emphasise that the metric represented by the tree can be used to quantify functional similarities.

#### The Functional Tree as Metric Model

The functional distance *D*_*f *_defined from the Functional Tree allows a new quantitative analysis of the functional relationship between gene products. To assess *D*_*f *_as a functional similarity measure we correlated sequence and annotated function similarity over a set of aligned pairs of yeast proteins. The benchmark set has been selected by applying a very restrictive criterion to obtain a high reliable set of annotated proteins. The selection process (see Methods section) takes as quality assay the evidence codes in GOA. In this work, we picked only those sequences that had been functionally characterized either by experimental assay (IDA evidence code) or by traceable published works (TAS evidence code) and whose GO terms were included in our functional tree.

**Figure 5:**
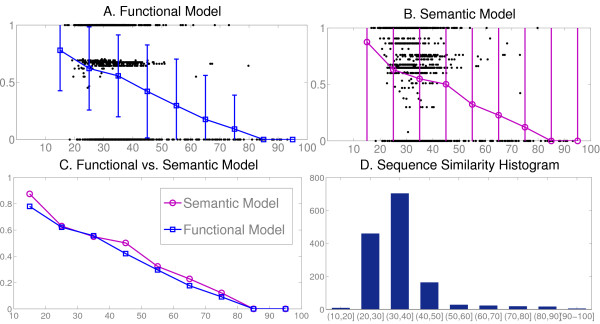
Comparison between functional distance and sequence similarity for pairs of Yeast proteins annotated with TAS and IDA evidence codes. The alignments covers most of the range of sequence similarities, whose distribution is shown in panel D. (A) Hausdorff distance (calculated using our functional metric) vs. sequence identity. The mean and the deviation values for each interval are also shown. (B) Hausdorff distance calculated using Lord's Semantic Similarity vs. sequence identity. (C) Mean values for both distance metrics. (D) Distribution of the percentage of Yeast protein pairs in each sequence similarity category.

The functional distance between proteins (through their sets of annotated terms) is calculated using the hausdorff definition. The details are exposed in the 'Functional Comparison between Gene Products' subsection from Methods. The distance values are represented against the sequence similarity (Figure 5A). Lord semantic similarity *D*_*s *_was also implemented and represented in Figure 5B. Note that the Lord distance values are normalised in order to analize the metric derived in this work with respect to Lord's model.

To compare the models the mean distance values for each bin of sequence identity are superposed in figure 5C. In average, both approaches correlate well with sequence similarity and exhibit a similar trend for homologous pairs. This is partly due to the homology-based mechanism of annotation that transfers directly a source set of MF-GO terms to many homologous sequences.

In consequence, more than 84% of the alignments with sequence similarity values greater than 80% share the same annotations. This lack of richness in the annotations limits further analysis of the methods. However, we can observe that *D*_*f *_and *D*_*s *_show a different behavior. The distance space is discretized into three well-defined groups (Figure 5A) whereas the semantic similarity values produced a great spread.

**Figure 6:**
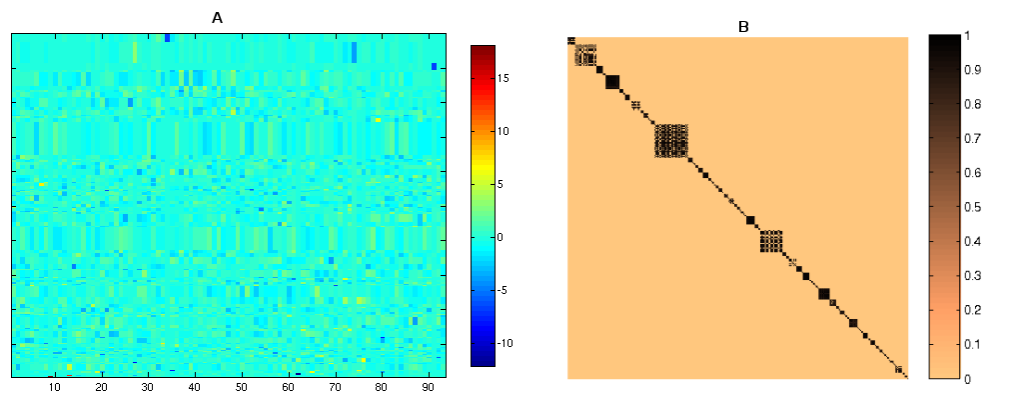
(A) Similarity Matrix in spectral space. The rows of the matrix represent the MF-GO terms in the reduced space of dimension 93. The terms are stacked in the same order that the Functional Tree (B) Ordered Similarity Matrix. The matrix was packed according to the optimal clustering. Each diagonal block correspond to a group in the Functional Tree. This matrix is close to an ideal block diagonal matrix (correlation coefficient of 0.86) that reveals a compact structure of functional groups.

These natural 'cut-offs' allow classifying the pairs into three categories with biological meaning that can be roughly labelled as 'closely functionally related' (distances less than 0.1), 'not related at all' (more than 0.9) and 'divergence in functionality' (in the intermediate interval with distances between 0.5 and 0.7). This partition results from the structure of the clusters (Figure 6B) showing small intra-group and large intergroup distances. This is in part due to 'biological' reasons but is also affected by the function transfer by sequence homology. 

The repetitive and persistent presence of the same MF-GO terms in the Intepro domains indicates clear functional associations of the terms but it is also originated by the usage of a reduced set of annotations producing redundancy in the functional information of the sequences and low coverage with respect to the total number of terms in the ontology (1532 from a total of 7417 MF-GO terms).

**Figure 7:**
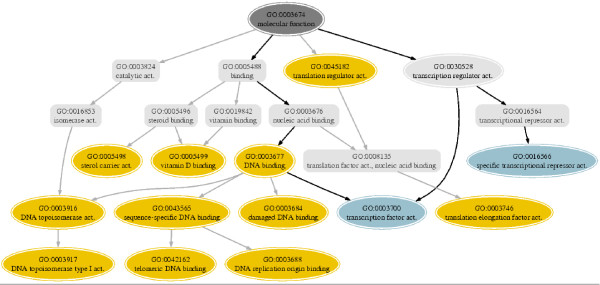
Graph representation of the ontology relations of a subset of MF-GO terms belonging to 'group 3' of the Functional Tree (orange nodes). The nodes in blue (GO:0016566 and GO:0003700) correspond to members of the 'group 3' that are also annotations of the pair [Uniprot:P20134]/[Uniprot:P10961]. The paths that links them are highlighted in black. Note there are two paths that connect them, and the least common ancestor is the node GO:0030528 ('transcription regulator activity') one level down from the root node. The Semantic Distance of the protein pair [Uniprot:P20134]/[Uniprot:P10961] is 0.76 whereas the Functional Distance is close to 0.

In addition, the Functional Distance Model *D*_*f *_becomes very useful from the perspective of recovering proteins functionally similar to a query, as it provides new associations between the terms inferred from the homology information in the database entries. These new links enrich the ontology relationships among the terms. This is the case of group 3 (analized in the 'Functional Groups' from Testing section) whose MF-GO terms are spread across different lineages of the ontology involving DNA-related activities. These associations are very visible in many Pfam domains (Hormone receptor, Sigma-70 factor, Ets-domain, HSF-DNA binding, GATA-type transcription activator etc ...) but are not detected with a criteria based on the semantic proximity of the terms. Group 3 is partially represented in Figure 7 showing the relations of some terms in the ontology. Some terms of the group, such as 'DNA binding' and 'specific transcriptional repressor activity', are very distant in the DAG and share the root as common ancestor. This produces a semantic distance of 1. Other terms like 'transcription factor activity' and 'DNA replication origin binding' share the node 'DNA binding' that is three levels apart from to the root.

The benchmark set includes some example pairs in which *D*_*f *_assigns close distances to functionally related pairs while *D*_*s *_does not. One is the pair formed by [Uniprot:P20134] and [Uniprot:P10961] that shares 50% sequence identity. The first is a transcriptional repressor and activator annotated with the term GO:0016566 ('specific transcriptional repressor activity'). The second is a trimeric heat shock transcription factor annotated with GO:0003700 ('transcription factor activity'). Both are characterized by HSF-type DNA-binding Pfam domain. The relative posititions of their annotated terms in the DAG can be checked in Figure 7. *D*_*s *_is 0.76 indicating a weak relation between the proteins. However, *D*_*f *_situates the pair into the 'closely functionally related' region because the terms belong to the cluster 3 described before.

Other similar example is the pair formed by the protein kinases [Uniprot:P32801] and [Uniprot:P41808]. The proteins are characterized by protein kinase pfam domain and are annotated respectively with GO:0004674 ('protein serine/threonine kinase activity') and GO:0004707 ('MAP kinase activity'). Both terms belong to group 6 (Functional Groups subsection). So, as in the example before, the distance *D*_*f *_is 0 whereas *D*_*s *_is 0.6. Although these terms are close in the ontology ('protein serine/threonine kinase activity' is ancestor of 'MAP kinase activity' and separated only by two depth levels), the Lord model assigns such a value distance because the shared parent ('protein serine/threonine kinase activity') is referred many times in the gene association.goa human file. According to Lord definition, high probable terms carry low information content producing high distance values in the comparison of terms. In the case of the kinases pair, the probability introduces a bias that shifts the semantic distance value to a region that indicates, as the example before, a weak relation between the proteins.

Finally, the Functional Distance model is sensitive enough to detect subtle differences in the pairs [Uniprot:P15700]/[Uniprot:P07170] and [Uniprot:P15700]/[Uniprot:P26364] that are explained by sequence analyses. In both cases, the members of the pair were annotated with GO:0004849 ('uridine kinase activity') and GO:0004017 ('adenylate kinase activity') respectively. Lord's Semantic Model produced a distance of 0.37 between these proteins, indicating semantical relation. In fact, the aforementioned terms are close in the GO hierarchy, and the deepest common parent shared by both GO terms, two levels above, is 'nucleobase, nucleoside, nucleotide kinase activity' (GO:0019205). However, our Functional Distance located that pair of GO terms within the intermediate interval at a distance of 0.65. A thorough analysis of the sequences revealed that [Uniprot:P15700] has the 'ADK' Pfam domain. Adenylate kinases are phosphotransferases with well conserved ADK domains that include an important arginine which inactivates the enzyme if mutated, and an aspartate that is located in the catalytic cleft and that forms a crucial salt bridge. However, in the particular case of [Uniprot:P07170] and [Uniprot:P26364], the putative ADK domain is interrupted by another PFAM domain, the ADK lid. Looking at the sequence of this particular region, the ADK domain boundaries were not clearly delineated due to a high degree of divergence in the active site. So, in this example our metric is able to capture the 'functional difference' between these two proteins due to the inserted domain.

### Implementation

The Spectral Clustering algorithm is implemented in Matlab 7.4.0 using the clustering functions available in the Statistics Toolbox. Lord's model is implemented in Python 2.5, and Python was also used to calculate the functional and semantic distances.

## Discussion and Conclusion

Here, we propose a new method to derive 'functional distances' between GO terms based on the co-occurrence of them in the same set of proteins. The simultaneous occurrence of terms in Interpro entries provides a natural biological link between the GO functions. The relationship between terms in the GO structure provides additional semantic information that helps to refine the metric model.

In this method, an initial profile is constructed for each GO term representing its association with a set of Interpro domains (after expanding the Interpro annotations with the parenthood relationships of the GO terms). These profiles are used to generate a matrix of co-occurrence between GO terms. A graph is constructed where the nodes are the GO terms and the edges are weighted according to the distances extracted from this co-occurrence matrix. Spectral clustering is applied to this graph in order to obtain an optimal number of groups of functionally similar GO terms. The distances derived in this way provide a hierarchical clustering of GO terms (functional tree) where the groups of terms with similar biological meaning tend to be close. Additionally, this 'Functional Tree' represents a metric model *D*_*f *_whereby the distances between the terms fulfil the mathematical properties of a metric space.

The main difference of this method from previous approaches [10-14] is that *D*_*f *_is learned from examples. Hence, in contrast to other proposed methods that derive the distances from the semantic relationships within the GO ontology, our method provides new associations between the terms that enables a different way to compare proteins in functional terms. We have selected some cases to illustrate this point, for which the functional similarity between related proteins is better estimated with this metric model than with currently available algorithms, such as Lord's 'Semantic Similarity Model' [10]. Moreover, we also tried to qualitatively assess some of the groups automatically extracted from the distances by a clustering algorithm. Over and above the comparison with these examples and the qualitative assessment of the functional tree, it is actually difficult to assess the general quality of any functional metric.

The overall representation of the functional distances in the Functional Tree originates very compact groups of terms separated by well defined intervals as it is shown in Figure 6B. This structure of the clusters produces a not uniform distribution of distances because the values tend to concentrate in three regions (low, intermediate and high), which is obviously a problem since it makes the metric to some extent 'qualitative'. On the other hand, this categorisation produces natural 'cut-offs' and functional similarities that are naturally classified by the method in three categories with biological meaning in a totally unsupervised way as been discussed in Results section.

This discretization is in part due to 'biological' reasons but is also affected by the homology-based transfer that causes that only a reduced set of terms is used for annotation purposes. In consequence, the coverage with respect to the total number of terms in the Molecular Function ontology is low (around 20%). In addition, only clear relations are selected resulting a set of 995 MF-GO terms that are considered in the Functional Distance Model.

It is important to note that the relationships between the GO terms obtained and the relationships in the GO ontology represent different elements. The GO DAG represents qualitative semantic relationships ('is-a' and 'part-of') while our relationships represent quantitative 'functional distances'.

Thus, the metric proposed here provides a way of quantifying how similar two functional annotations are. This can be very useful for training systems for function prediction, for function-based protein searches in databases, or to assess the accuracy of a functional prediction (comparing the predicted set of annotations with the real one). This metric could also be useful for analysing the gene clusters produced by DNA array experiments. We think it may also provide insights into how functions evolved and the relationships between sequence, structure and functional spaces.

## Methods

### Similarity Matrix

The GO annotations for a given Interpro entry are retrieved from the mapping of Interpro to Gene Ontology [23] (interpro2go file, release May 2006). Only the GO terms belonging to the MF-GO are considered.

For each MF-GO term a profile vector is created that describes the presence/absence of the terms throughout the database. The profiles are constructed to analyze the simultaneous occurrence of pair MF-GO terms in the Interpro entries and filter the cases that do not contribute to the extraction of the relationship between the terms. The similarity between two terms is calculated by the cosine distance between their co-occurrence vectors:

(1) $$Sim(GO_{i},GO_{j})=cos(\vec{P_{i}},\vec{P_{j}})=\frac{\vec{P_{i}}*\vec{P_{j}}}{\left|\vec{P_{i}}\right|\left|\vec{P_{j}}\right|}$$

Note that the cosine distance generates values ranking between 0 and 1. The similarity value can be considered as a description of the functional relationship between these terms, whereby similarities equal to 0 stand for unrelated terms and 1 stands for strongly related. The similarity matrix *S *is plotted in Figure 2A together with its histogram (Figure 2B).

The distribution of the similarity values shows that almost 90 per cent of the pairs of terms are only weakly related. This structure of the relationships reflects the presence of well-defined groups of terms. However, the search space has been limited as the cosine distance assigns a non-zero value even to pairs of terms that rarely share the same Interpro entry. Thus, based on the inspection of the histogram, we set a threshold of 0.8 to select strong functional links. In total there are 995 MF-GO terms that are significantly connected.

Additionally, we reduced the dimension of *S *by applying a NJW Spectral Clustering (NJW-SC) algorithm [21]. The similarity matrix in spectral space is shown in 6A, while the details of the algorithm are outlined in the 'Spectral Clustering Algorithm' subsection from the appendix.

### Optimization Approach

As the number of clusters K is not initially known, an optimization approach is used, such as the multiway normalized cut value *MNCut*, (see 'Multiway Cuts' subsection from the appendix). The *MNCut *was calculated from the normalized matrix *P *(Transition Probability matrix). *P *represents the total probability of transit between any two clusters *C*_*i *_and *C*_*j *_for a given partition *C *= {*C*_1 _... *C*_*K*_} of the graph and *MNCut *and represents the total sum of the transition probabilities between the clusters. The goal of optimization is to find the eigenvalue cut-off that generates a partition C* (*K*) that minimizes the *MNCut *value. In particular, we addressed optimization by exploiting the minimization of the *gap *value over the spectra of *S*.

**Figure 8:**
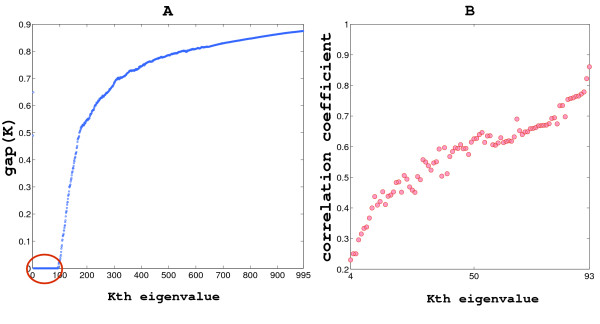
The whole spectra of the P matrix [λ_*i*_(*P*)]_*i*∈*U *_is analyzed selecting the first K eigenvalues and for each selection obtaining a partition of the MF-GO terms *C*_*K*_. In panel A, the values of the gap measure calculated for *C*_*K *_are represented and according to the Spectral Clustering theory, the best partition C* minimizes the gap value. The red circle encloses the eigenvalues of the spectra that generate 'good' clusterings (interval [4, 93]). Panel B shows the result of applying a second criterion to select the best number of groups from the interval. The correlation coefficient of the ordered similarity matrix with an ideal block diagonal matrix is calculated for each partition. The best clustering is obtained by selecting the first 93 eigenvalues.

The optimization curve is shown in Figure 8A. Note that a wide range of eigenvalues minimizes the gap (from 4 to 93). Thus, we applied an additional criteria to select the optimal cut-off, the correlation coefficient between the *S *matrix packed according to C* (*K*) and the ideal block diagonal matrix for this partition. The correlation calculation is used as a measure of the 'compactness' of the each partition. The correlation coefficient values are shown in 8B where the partition for the 93rd eigenvalue maximizes the procedure (correlation of 0.86). In Figure 6B the *S *matrix packed for the optimal clustering is represented.

### Benchmark Dataset of aligned proteins

To compare our metric with others developed previously and to evaluate its relationship with sequence similarity, we took a set of proteins with reliable annotations taken from the Saccharomyces Genome Database (SGD). As an annotation source we used the file gene association.sgd (release October 2006).

The confidence of the Gene Ontology Annotations (GOA) is represented by the Evidence Code (EVC). Although there is no consensus rule that establishes a standard order of annotations based on the EVCs, the Gene Ontology Consortium has outlined a rank of EVCs as a guide [24]. The hierarchy of confidence establishes that the TAS (Traceable Author Statement) and IDA (Inferred from Direct Assay) tagged annotations offer the highest confidence.

Despite the efforts of the GOA project to improve the general reliability of its databases, the bulk of GO assignments are still made by automatic techniques with no expert curation. This homology-based transfer generates highly redundant sets of GO annotations. Moreover, as our method to derive the similarity between GO terms is to some extent affected by sequence similarity (given that it uses Interpro domains), we decided to exclude GO annotations derived from sequence relationships.

In gene association.sgd, there were 1264 yeast proteins annotated with GO terms with EVCs unrelated by homology (TAS and IDA) that also appeared in our functional tree. After filtering this set for sequence redundancy with CD-hit [25] at 95%, we obtained a final set of 1193 yeast proteins. We then perform fast alignments of all-against-all using BLAST, having chosen a permissive e-value (0.1) to permit alignments between distant sequences. Nevertheless, alignments covering less than 50 residues and/or with less than 10% similarity were excluded. The final set comprised 1426 protein pairs and the distribution of sequence similarity for these pairs is shown in Figure 5D.

### Functional Comparison between Gene Products

To calculate the functional similarity between two proteins from their set of GO terms and the metric relating these terms, we applied the Hausdorff Distance. The Hausdorff Distance is defined as the maximum value between any point within one set and the nearest point in the other set. Formally, from set A to B is:

(2) $$D_{hausdorff}^{a\to b}=\underset{a\in A}{\max}\{\underset{b\in B}{\min}(D(a,b))\}$$

As the Hausdorff Distance is not symmetrical, a symmetrical measure was formulated as:

(3) $$D_{hausdorff}=max(D_{hausdorff}^{a\to b},D_{hausdorff}^{b\to a})$$

Usually the Hausdorff distance is evaluated over the Euclidean space, although in this work we applied equation 3 using two distances: (A) the distance *D*_*f *_obtained from our Functional Tree and (B) the distance proposed by Lord et al. [10]

We implemented Lord's semantic similarity using as a reference the annotated database gene associa-tion.goa human [26] (released version 45.0), and we normalised the values between 0 and 1 to compare it with the metric derived in this work.

## Authors' contributions

All authors contributed to the development of the methodology. AP implemented the algorithm and evaluated the method for the alignment set. FP analyzed and interpreted the functional groups. AP and FP contributed to writing the manuscript, and all the authors read and approved the final manuscript.

## Appendix

### Spectral Clustering

Spectral clustering has its origin in spectral graph partitioning [27] and is intended to efficiently identify good discrete partitions of a graph based on the eigenvalues and eigenvectors of the Laplacian matrix of the graph.

Spectral clustering belongs to a collection of techniques that are designed to overcome the problems of previous approaches by using new ideas such as the eigenvectors of the generalised/normalized Laplacian or the multi-way spectral cut. A systematic comparison between the existing published algorithms can be found in the work of Verma and Meila [20]. Therein, the authors present a clear description of the basic steps of the algorithms and their general classification based on three different strategies: (I) recursive spectral; (II) multi-way spectral; and (III) non-spectral.

In this section we will introduce the notation and the basic steps for the NJW spectral clustering algorithm [21] and the ideas behind multi-way spectral cuts as a criterion of optimisation to find the best partition of the data. Here we implemented a modified version of NJW algorithm suggested in [20].

### NJW Spectral Clustering Algorithm

Consider a dataset *U *formed by N points to be clustered. For each pair of points within *U*, a similarity value can be defined as *s*_*ij *_= *s*_*ji *_≥ 0 by any similarity measure. *U *can be represented by a weighted directed graph *G *= (*V*, *E*) where the *S *= [*s*_*ij*_] matrix plays the role of the adjacency matrix of the graph. A clustering *C *= *C*_1_, *C*_2_,..., *C*_*K *_is a partitioning of U into non-empty disjointed subsets *C*_1_, *C*_2_,..., *C*_*K*_.

The out-degree of a node *j *is defined as $d_{i}=\sum_{j=1}^{N}s_{ij}$. We represent *D *for a diagonal matrix of out-degrees as: *D *= *diag*(*d*_1_,..., *d*_*N*_).

The nodes can be grouped by following the steps:

1. Compute the transition probability matrix *P *= *D*^-1 ^*S*.

where *P *defines the probability to navigate from node *i *to node *j *in a random walk over *G*. By construction, the eigenvalues of *P *are delimited as [-1, 1], 1 = *λ*_1 _≥ *λ*_2 _≥ ⋯ *λ *_*N *_≥ -1. The corresponding eigenvectors are *v*^1^,..., *v*^*N*^.

2. Select the first *K *eigenvalues of *P *and form the *X *matrix by stacking the eigenvectors in columns:

*X *= [*v*^1^*v*^2^...*v*^*K*^]

3. Normalize each row of *X *to unit length to form the *Y *matrix:

$$Y_{ij}=\frac{X_{ij}}{\sqrt{\sum_{j}X_{ij}^{2}}}$$

4. Treat each row of Y as a point in *K *dimensions. The points can be grouped by any standard clustering technique. In this work, the points are organized in a hierarchical tree.

### Multiway Cuts

The majority of approaches in spectral clustering deal with partitioning the graph in two optimal parts by using one eigenvector at a time and applying this approach reiteratively until *K *clusters are found.

A way to use the *K *first eigenvectors simultaneously to find the optimum partition of the graph has been proposed, minimizing the cut of two partitions over all possible partitions in *U *[28]. Most of the approaches assume that the number of clusters *K *is known in advance, but in many problems related to clustering there is not indirect evidence that reveals the optimal number of groups.

Here we expose the basics of the multi-way normalized cut (*MNCut*) concept that has been applied to find the optimum number *K*:

The volume of node *i *is defined as the out-degree of the node:

$$D_{i}=Vol\{i\}=\sum_{j\in U}S_{ij}$$

*D *denotes the diagonal matrix formed by *D*_*i*_. The volume of a subset *A *⊆ *U *is $VolA=\sum_{i\in A}D_{i}$, (we assume that no node has volume 0).

Given two disjoint subsets (*A*, *B*) ⊂ *U*, the set of edges between the subsets is the *cut *between *A *and *B*:

$$Cut(A,B)=\sum_{i\in A,j\in B}S_{ij}$$

and the probability of transit from set *A *to set *B *is:

$$P_{AB}=\frac{Cut(A,B)}{VolA}$$

Given the partition *C *= {*C*_1_,...*C*_*K*_}, over *U *the multi-way normalized cut clustering criteria introduced in [28] is defined as:

The multi-way normalized cut represents the total sum of the transition probabilities between the clusters of *C*. If *MNCut*(C) is small then the probability of evading *C*_*k *_in a random walk is also small.

It has been shown that for any clustering, the *MNCut*(*C*) is low-bounded by a function of the number of clusters *K *and the eigenvalues of *P *[29]:

$$MNCut(C)\geq K-\sum_{k=1}^{K}\lambda_{k}(P)$$

The non-negative difference between the *MNCut*(*C*) and its lower bound is the *gap*(*C*):

$$gap(C)=MNCut(C)-K+\sum_{k=1}^{K}\lambda_{k}(P)$$

It has been also shown that *gap*(*C*) is 0 if P has piecewise constant eigenvectors, that is if *P *is an ideal block stochastic matrix [29].

Therefore, from a set of *M *different partition solutions of the data [*C*_*i*_]_*i*∈*M*_, the optimal C* is that which minimizes the *gap *measure.

### Properties of a Metric Space

By construction, the hierarchical clustering procedure over the Gene Ontology terms defines a generalized distance *D *between any two terms *go*_*a *_and *go*_*b *_that satisfies the mathematical properties of a metric space [22]. That is, any set of elements (terms) of the space *x*_1_, *x*_2 _and *x*_3 _fulfil:

• Nonnegativity: *D*(*x*_1_, *x*_2_) ≥ 0

• Reflexivity: *D*(*x*_1_, *x*_2_) = 0 if and only if *x*_1 _= *x*_2_

• Symmetry: *D*(*x*_1_, *x*_2_) = *D*(*x*_2_, *x*_1_)

• Triangle Inequality: *D*(*x*_1_, *x*_2_) + *D*(*x*_2_, *x*_3_) ≥ *D*(*x*_1_, *x*_3_)

## Supplementary Material

Additional file 1 Functional Tree. The data provided represent the 'Functional Tree' joining the Molecular Function Gene Ontology terms. Click here for file
